# Between the classic hit and the modern sequel: transition dosing between peginterferon alfa-2a and ropeginterferon alfa-2b for myeloproliferative neoplasms

**DOI:** 10.1007/s00277-026-07143-5

**Published:** 2026-06-24

**Authors:** Ghaith Abu-Zeinah, Gabriela Hobbs, Brandi N. Reeves, Abdulraheem Yacoub, Joseph M. Scandura

**Affiliations:** 1 Richard T. Silver MPN Center and the Division of Hematology & Medical Oncology, Weill Cornell Medicine, New York, NY USA; 2 Mass General Brigham Cancer Institute, Harvard Medical School, Boston, MA USA; 3 Lineberger Comprehensive Cancer Center and the Division of Hematology, University of North Carolina at Chapel Hill, Chapel Hill, NC USA; 4 Hematologic Malignancies and Cellular Therapeutics, University of Kansas Cancer Center, Westwood, KS USA

Dear Editor,

As previously noted by Mesa et al. [1], the limited availability of Peginterferon alfa-2a (PEG) confirmed by the FDA in January 2025, has impacted many patients with myeloproliferative neoplasms (MPNs) relying on off-label use of PEG for long-term disease control. The limited availability of PEG was a result of the change of the marketing authorization holder and the introduction of a new manufacturing site. As of March 2026, the regulatory assessment for the manufacturing site approval in the US is still ongoing but PEG remains available. Ropeginterferon alfa-2b (ROPEG), approved by the FDA for polycythemia vera (PV), is a viable alternative for some of those impacted by the limited availability of PEG. With both formulations in use, transitioning between PEG and ROPEG remains an important question when supply or cost coverage issues arise. However, there are no prospective studies to determine the optimal dosing when transitioning between interferon-α (IFN) formulations. Real-world evidence (RWE) is also lacking and limited to case reports, making it difficult to reach consensus on a transition dosing strategy. Herein, we provide our perspective on IFN dosing when transitioning between PEG and ROPEG.

IFN is a disease-modifying treatment for MPNs that can target malignant stem cells [2], and induce durable clinical and molecular responses with improved event-free survival for patients with polycythemia vera (PV) and essential thrombocythemia (ET) [3–5], and reverse marrow fibrosis in patients with myelofibrosis (MF) [6]. IFN is currently the preferred or first-line cytoreductive option for many patients with ET and PV [7] and is a standard of care at our centers. At Weill Cornell Medicine (WCM), IFN use in MPNs dates back to the 1980s [8], and currently 42% of WCM patients with polycythemia vera (PV) are treated with interferon (IFN) [4]. This adoption of IFN has been linked to improved survival of patients with PV, and potentially normal life expectancy [9]. A smaller but significant proportion of essential thrombocythemia (ET) and myelofibrosis (MF) patients at WCM are also treated with IFN (24% for both). The use of IFN has expanded beyond our academic centers, particularly since the approval of ROPEG in the US in 2021, marking the importance of clinical experience with IFN dosing when transitioning between formulations.

The widely adopted dosing approach for PEG is a low initial dose, generally 45 mcg weekly with response-based titration (LDRT), in 45 mcg increments [10]. LDRT was adopted because high starting doses of non-pegylated IFN and PEG [11] were poorly tolerated by patients in earlier studies. LDRT PEG has been effective in our hands and others, achieving a complete hematologic response rate (CHR) over 50% and overall response rate (ORR) over 90% at 1 year, and yielding a low discontinuation rate of 2.3%-6.5% [12–14]. On the other hand, ROPEG has two dosing approaches adopted currently: (1) on-label LDRT approach starting with 50–100 mcg every two weeks and up titrating in 50 mcg increments, and (2) the investigational HiDAT approach or high starting dose (250 mcg) with accelerated titration (to 350, and 500 mcg in two-week intervals) [15]. One HiDAT study [16] reported a median time to CHR of 22.5 weeks, potentially earlier than anticipated by the LDRT approach on PEGINVERA (34 weeks) and PROUD/CONTI-PV (> 52 weeks) [17, 18], but whether this holds in head-to-head comparison is an issue under investigation (the ECLIPSE-PV trial, NCT05481151) [19]. The more important question of whether rapidity of response improves event-free survival outcomes will need to be evaluated.

In patients transitioning from PEG to ROPEG, we implement an LDRT approach since results of the ECLIPSE-PV trial are not yet available to determine if HiDAT speeds CHR or causes excess toxicity. If a transition is required for patients on PEG, we generally transition to ROPEG at the starting dose of 100 mcg every two weeks, uptitrated by 50 mcg increments every 2 to 4 weeks as needed to maintain response after switching. Higher doses for transition risks adverse events that may jeopardize treatment continuity such as dose-dependent IFN class-related symptoms, or asymptomatic but significant lab abnormalities (transaminitis, cytopenias) that necessitate treatment hold. This careful approach also considers the unlikelihood of a universal dose conversion between PEG and ROPEG owing to differences in pharmacokinetic/ pharmacodynamic profiles, variability in IFN metabolism and clearance between patients, and disease heterogeneity.

The transition for those on higher PEG doses for maintenance (e.g. 135–180 mcg weekly) to ROPEG is perhaps more complex where both LDRT or HiDAT are viable options balancing tolerance or efficacy respectively [20]. In such cases, a PEG to ROPEG transition with a HiDAT approach is reasonable for those in partial or suboptimal response when transitioning but not necessarily for those in CHR. Alongside hematologic response, molecular response assessment may have a role in dose selection and modification but such molecularly-guided dosing would need to be rigorously evaluated. Similar considerations apply for a ROPEG to PEG transition, which may arise in the context of cost coverage rather than limited availability.

There is currently no evidence to indicate PEG and ROPEG would have different outcomes, but dosing may be relevant for efficacy and tolerability. In a propensity-score matched analysis of 44 ROPEG and 44 PEG patients with PV using LDRT dosing approach, there was no difference in 1–2-year outcomes pertaining to CHR, ORR, adverse events, and event-free survival (EFS) [12]. Only a single disease-related event (deep vein thrombosis) was experienced (~ 0.5 per 100 patient-years). Although this study did not examine outcomes of PEG to ROPEG or ROPEG to PEG transitions, it identified short-term safety and similar efficacy of both formulations at LDRT, thereby supporting interchangeability at lower dose levels.

In conclusion, thoughtful dosing and transition strategies between PEG and ROPEG will be essential to ensure continuity of efficacy and safety of IFN in real-world practice. Without a head-to-head comparison, the choice between ROPEG and PEG continues to be based on availability, cost coverage, and individualized preference such as dosing convenience. If transitioning between formulations, we favor a similar LDRT dosing approach for both PEG and ROPEG, until a demonstrable benefit of HiDAT ROPEG on clinically important outcomes such as thrombosis, progression or death can be shown. A higher transition dose than standard LDRT can be considered in those with suboptimal response but tolerant to their IFN formulation. If PEG to ROPEG transition (or vice versa) is required for shortage, cost/ health insurance issues, or perhaps tolerability, maintaining an LDRT approach for those already on low dose prioritizes safety/ tolerance. Additional RWE is needed to determine the optimal transition dosing approach, while understanding the need for individualized dose adjustments.

## Data Availability

No datasets were generated or analysed during the current study.
